# 

**Published:** 1879-11

**Authors:** 


### Table of Contents.

### ORIGINAL COMMUNICATIONS.

Exsection of Sup. Maxillary Nerve. By Herbert W. Little, M.D., New York....... 449
Fibroid Uterus. By Herbert W. Little, M.D., New York....... 451
High-Land Malarial or Mushroom Fever. By J. G. Westmoreland, M.D., Atlanta, Ga....... 453

### REPORTS OF SOCIETIES.

Atlanta Academy of Medicine....... 456
Chicago Medical Society....... 460
New York Pathological Society....... 462

### SELECTIONS.

The Pathology of, and the Treatment of Ulcers, Varicose Veins and Cutaneous Diseases of the Leg by Martin's Pure Rubber Bandage....... 472
Electricity a Paralyzing Agent....... 479
A Case of Carcinoma Ventriculi....... 489
A Case of Double Vagina and Cervix Uteri....... 492
The Philosophical Treatment of Diphtheria....... 497

### EDITORIAL.

Do Nostrums Benefit Consumers?....... 506
A Word With Our Readers....... 507
Editorial Correspondence....... 508

### BIBLIOGRAPHICAL.

A Clinical Treatise on the Diseases of the Nervous System....... 511
Diseases of Women....... 511
Analysis of the Urine with Special Reference to the Diseases of the Genito Urinary Organs....... 512
The Multum in Parvo Reference and Dose Book....... 512

### EXCERPTA.

Nichol's Elixir....... 512
Phosphorole....... 512

